# Erroneous intra-articular injection of gadolinium solution at 0.5 mol/l concentration: a case report

**DOI:** 10.1186/1757-1626-2-9320

**Published:** 2009-12-14

**Authors:** Eugenio A Genovese, Elena Bertolotti, Carlo Fugazzola

**Affiliations:** 1 Department of Radiology, Insubria University, Via Guicciardini, 21100 Varese, Italy

## Abstract

Magnetic Resonance arthrography is considered the gold standard imaging technique for the study of shoulder instability and tendon tears.

We describe an image artefact, characterized by decreased signal intensity of the paramagnetic gadolinium chelate contrast agent during a shoulder Magnetic Resonance arthrography, attributable to an incorrect concentration which does not cause evident capsular damage and is completely absorbed after 48 h from the administration.

## Background

Magnetic resonance (MR) arthrography is the gold standard imaging diagnostic technique for shoulder joint examination, mainly in the detection of elementary lesions causing shoulder instability and tendon tears [1].

The first time of this technique consists in arthrography with direct injection of contrast agent into the joint with or without fluoroscopic guidance. In the second time of the procedure, MR examination is performed by various sequences, orientated on axial, oblique coronal and sagittal planes.

The paramagnetic contrast agent used commonly is the dimeglumine salt of gadopentetate acid (Gd-DTPA) in different dilutions with saline solution.

During MR arthrography of the shoulder, we found an image artefact, characterized by decreased signal intensity of the contrast agent injected into the joint, caused by incorrect concentration.

To the best of our knowledge such an effect, using paramagnetic contrast agent not diluted with iodinated contrast solution in MR arthrography of the shoulder, has not been reported in vivo yet.

## Case presentation

A 39-year-old Caucasian male, complaining about chronic non traumatic shoulder pain, with non-significant radiological and ultrasound examinations, was referred to our department to perform MR arthrography.

Our protocol includes the use of pre-filled syringes containing dimeglumine salt of gadopentetate acid diluted in saline solution (0.9% sodium chloride) at 2 mmol/l concentration (Magnevist^© ^2 mmol/l, Schering, Berlin, Germany).

After the patient had signed informed consent, we performed the first time of the examination injecting contrast agent by anterior approach to shoulder joint, without fluroscopic guidance; we injected 20 cc of contrast agent and we found an increased resistance at capsular filling during the administration.

In the second time of our MR arthrography protocol, we performed, with a surface coil, SE T1-weighted sequences on axial, oblique coronal and sagittal plane, and a TSE T2/PD sequence with fat saturation technique on oblique coronal plane, using a Intera Tomograph 1,5-Tesla (Philips, Koninklijke, Netherlands).

**Figure 1:**
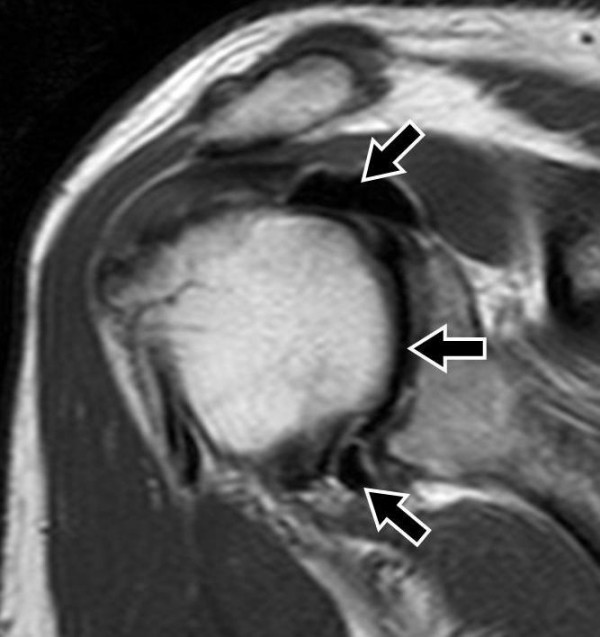
**SE T1-weighted sequence (TR/TE/Thick: 500 ms/10 ms/4 mm) on oblique coronal plane after administration of dimeglumine salt of gadopentetate acid diluted in saline solution at 0.5 mol/l concentration**. Artefact is detectable, characterized by contrast signal absence (black arrows), with no evidence of contrast agent in peri-articular soft tissues.

The SE T1-weighted sequence on oblique coronal plane is useful to display that contrast intra-articular injection is technically correct, showing capsular filling and not contrast agent in the peri-articular soft tissues. On the contrary, we found out an image artefact characterized by signal absence in the contrast solution injected in the shoulder, instead of the hyperintensity of contrast signal expected in ideal conditions (Figure 1).

Our examination did not cause immediate or late side effects and the patient did not complain about any decrease of shoulder motility or increase of pain.

Another MR arthrography was then performed about 48 h later using pre-filled syringes containing dimeglumine salt of gadopentetate acid diluted in saline solution (0.9% sodium chloride) at 2 mmol/l concentration as our protocol includes.

**Figure 2:**
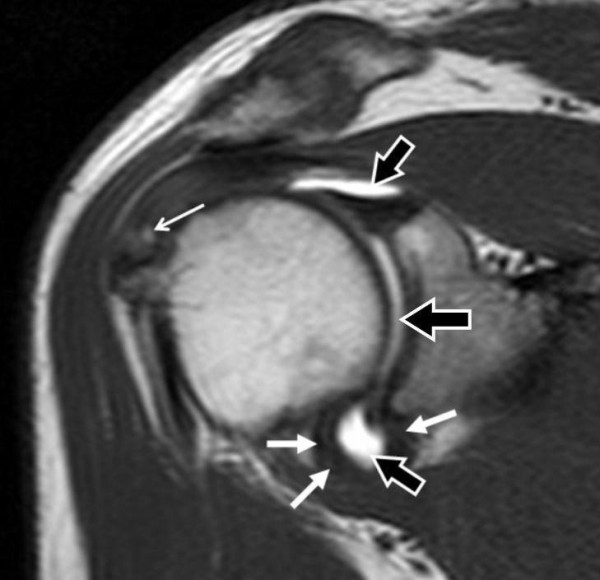
**SE T1-weighted sequence (TR/TE/Thick: 500 ms/10 ms/4 mm) on oblique coronal plane, after administration of dimeglumine salt of gadopentetate acid diluted in saline solution at 2 mmol/l concentration**. The expected contrast signal hyperintensity is detectable (black arrows). Capsular wall fibrous thickening at axillary pouch (white arrows) and supraspinatus tendon partial tear on articular side (point white arrow) are evident.

**Figure 3:**
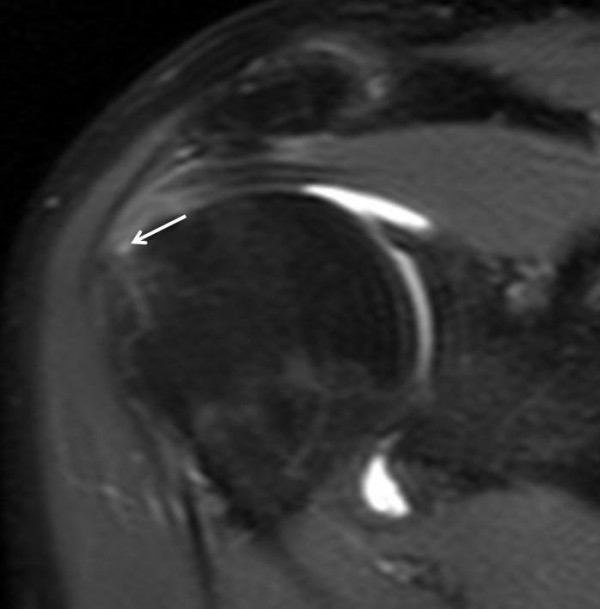
**Proton Density FS-weighted sequence (TR/TE/Thick: 4011 ms/7,272 ms/4 mm) on oblique coronal plane, after administration of dimeglumine salt of gadopentetate acid diluted in saline solution at 2 mmol/l concentration**. Supraspinatus tendon partial tear on articular side was confirmed (point white arrow).

The new examination showed the expected hyperintensity of contrast agent signal (Fig. 2) as in ideal conditions and the disappearance of the previous image artefact characterized by signal absence. We were then able to evaluate intra-articular structures and to answer clinical question, detecting supraspinatus tendon partial tear on articular side and fibrous thickening of the capsular wall of the axillary pouch, as a sign of adhesive capsulitis (Figure 2), The Proton Density Fat Saturation weighted sequence Supraspinatus confirmed tendon partial tear (Figure 3).

Our revision of the first MR arthography procedure disclosed that human mistake occurred in the choice of contrast agent solution; in fact, a pre-filled syringe with dimeglumine salt of gadopentetate acid diluted in saline solution (0.9% sodium chloride) at 0.5 mol/l concentration had been accidentally used. This error was facilitated by an external similarity of the two syringes.

No immediate or late side effects occurred even after the second MR arthrography.

## Discussion

Shoulder MR arthography is characterized by higher sensitivity than non enhanced MR in the detection of elementary lesions of rotator cuff tendons and capsulo-legamentous structures [1].

MR arthrography is performed with anterior approach to shoulder joint under fluoroscopic guidance or without it and the choice of the site of injection is made with specific anatomic criteria. Fluoroscopic guidance requires the addition of non ionic iodinated contrast solution to paramagnetic contrast agent to detect intra-articular placement of the needle [2]; on the contrary, in the procedure without fluorscopic guidance, such a solution is not necessary [3].

In our protocol we use pre-filled syringes with Gd-DTPA solution at 2 mmol/l concentration. In vitro studies [3] show that this is the ideal paramagnetic contrast agent concentration in order to evaluate the anatomic structures we aim to study. Paramagnetic contrast agents display a biphasic behaviour, with high signal/noise rate in T1 relaxation time and decrease in T2 relaxation time because the agents generally reduce the relaxation times of the tissues. In vitro studies show that even small amounts of iodinated contrast agents decrease significantly paramagnetic contrast intensity [2,3]. Such an effect was reported also in vivo [4].

In our case, Magnetic Resonance Imaging (MRI) obtained after paramagnetic contrast solution administration, without iodinated contrast agent, showed unusual contrast signal behaviour, characterized by signal absence in SE T1-weighted sequence. After a careful revision of the procedure, we detected a mistake in the choice of the pre-filled syringe, as a contrast solution prepared for intra-vascular administration and not for intra-articular injection was used.

The two solutions contained the same components at different concentration; in fact, the intra-articular contrast agent we used was at 2 mmol/l concentration while the one accidentally used in our case was at higher concentration and this provides a prevalent effect on T2 relaxation time, a rare event after intravenous injection, due to the dilution in the blood stream. Therefore the signal alteration described was caused by the abnormally high Gd-DTPA concentration and not by the presence of iodinated contrast, as reported by Montgomery and coll [4]. Aydingoz et al [5] described a similar signal alteration during a MR arthrography of the hip performed using an accidental excessive amount of gadolinium chelates diluted with saline solution and local anesthetic.

The combination with other substances could not influence the image artefact, as the Authors suppose, because we demonstrated that the local anesthetic acts like saline solution when it is mixed with Gd-DTPA [3]. So, we agree with the Authors when they state that another possible explanation could be the excessive intra-articular administration of the paramagnetic contrast agent.

In the case reported no immediate or late side effects occurred after high concentration of paramagnetic contrast administration: therefore we confirm that paramagnetic contrast does not cause clinically evident side effects as at dilution commonly used [6] as at the concentration routinely injected intravenously.

The subsequent MR arthrography performed in our case shows that the whole amount of contrast agent injected was absorbed by joint synovial tissue, with no contrast left-over.

We noticed an increased resistance at capsular filling during contrast solution administration and it was confirmed, even if less remarkable, in the subsequent MR arthography. This can be only partly justified by the different viscosity characterizing the two solutions; [2] in fact, in the following MR arthography we detected signs interpreted as adhesive capsulitis with decreased filling of the axillary pouch.

## Conclusion

In conclusion, intra-articular paramagnetic contrast agent not diluted with iodinated contrast solution does not cause evident capsular damage and is completely absorbed after 48 h from the administration.

## Abbreviations

Gd-DTPA: gadolinium diethylenetriamine penta-acetic acid; MR: magnetic resonance.

## Consent

Written informed consent could not be obtained because the patient was lost to follow-up. Despite repeated attempts we were unable to trace the patient or her family. We believe this case report holds a worthwhile clinical lesson which could not be communicated effectively in any other way. Every effort has been made to keep the patient's identity anonymous. We would not expect the patient or her family to object to publication.

## Competing interests

The authors declare that they have no competing interests.

## Authors' contributions

EB analyzed and interpreted the patient data regarding the shoulder's disease. EAG and EB performed the MR examination. EAG and CF were a major contributor in writing the manuscript. All authors read and approved the final manuscript.
